# Effect of Imidazoline Inhibitor on the Rehabilitation of Reinforced Concrete with Electromigration Method

**DOI:** 10.3390/ma13020398

**Published:** 2020-01-15

**Authors:** Chonggen Pan, Jianghong Mao, Weiliang Jin

**Affiliations:** 1 Ningbo Institute of Technology, Zhejiang University, Ningbo 315100, China; 2 Ningbo Research Institute, Zhejiang University, Ningbo 315100, China; 3 Institute of Structural Engineering, Zhejiang University, Hangzhou 310058, China; jinwl@zju.edu.cn

**Keywords:** reinforced concrete, durability, imidazoline inhibitor, chloride

## Abstract

Steel bars embedded in reinforced concrete are vulnerable to corrosion in high chloride environments. Bidirectional electromigration rehabilitation (BIEM) is a novel method to enhance the durability of reinforced concrete by extracting chloride out of concrete and introducing an inhibitor to the surface of the steel bar under the action of an electric field. During the migration process, a higher ionization capacity of the inhibitor with a symmetrical molecular structure was introduced. A new imidazoline inhibitor was, therefore, employed in this study due to its great ionization capacity. The effect of imidazoline and triethylenetetramine inhibitor on chloride migration, corrosion potential, and strength of concrete were explored. The research results showed that the effect of chloride extraction and electrochemical chloride extraction made no significant difference on the surface of the concrete, where chloride extraction efficiency was more than 70%, and the chloride extraction efficiency was more than 90% around the location of the steel. while a dry-wet cycle test, the potential of concrete increased by about 200 mV by mixing imidazoline inhibitor. The imidazoline inhibitor was found to be effective at facilitating chloride migration and ameliorating corrosion, meanwhile, it had a negligible impact on the concrete’s strength.

## 1. Introduction

Reinforced concrete is considered as one of the most widely used engineering materials for constructions, and its durability is the major problem affecting the service life of the engineering structures. Corrosion induced by chloride ions ingress damages the passive film by penetration [1], which is a major cause of the failure of the reinforced concrete structures. When rebar becomes corroded, the volumetric expansion rate of the rust ranges from 2.26% to 3.46%, which results in cracking of the concrete. Concrete in extreme environments, such as the sea and saline lake areas, frozen polar environments, and temperature differences experienced by desert regions, suffers from chloride ion erosion and easily becomes unserviceable early [2]. Australian researchers investigated the corrosion of coastal wharves and found severe corrosion of the steel bars [3]. In the Nordic countries, Canada, Australia, and many other countries, the corrosion of steel induced by chloride is a common occurrence in many construction projects. Therefore, large amounts of funding have been spent on the associated repair and replacement of materials [4]. In particular, China’s losses from marine environment corrosion account for 10% of the investment in construction engineering, steel corrosion was the main reason causing premature deterioration of reinforced concrete [5].

Various protective methods, including epoxy-coated steels, impregnation of inhibitor, are used to prevent corrosion in new structures. For old structures, a number of techniques have been studied and subsequently employed including cathodic protection, realkalisation and electrochemical chloride extraction (ECE). However, those methods cannot completely remove the chlorides in the structure and may therefore only be temporary solutions as chloride ions may remain available to attack the steel reinforcement once the treatment ends [6,7,8]. Bidirectional electromigration rehabilitation (BIEM) is an important method of improving the durability of reinforced concrete. This technique discharges chloride ions from concrete and moves the inhibitor into the vicinity of the reinforcement under the action of an applied electric field. Upon arrival at its destination, the inhibitor adheres to the surface of the steel through physical and chemical adsorption [9,10]. At present, the commonly used inhibitors for bidirectional electromigration rehabilitation are mainly amine, alcoholamines compounds. The electromigration inhibitor forms a protective film on the surface of the reinforcing steel bar and blocks the pores. It can reach the reinforcement surface through pores in the concrete through siphonage and electric fields, where the non-polar group of the inhibitor arranges on the reinforcing steel surface to form a hydrophobic layer. This layer of protection can prevent metal ions and corrosion medium, water, oxygen from penetrating to the metal surface, thus playing a role of preventing rust and ‘second corrosion’ [11,12]. When the inhibitor reaches a certain concentration on the surface of the reinforcing steel, it will form a dense protective film that can separate the corrosive medium, such as chlorine ions and oxygen, from the steel bars as a mean of preventing destruction [13]. Electrolysis occurs when ions are transferred. And amounts of OH^-^ are produced on the surface of the reinforcement, which improves local basicity and is beneficial to the passivation of the steel bar [14,15].

The applicability of organic compounds as corrosion inhibitors for the metals in acidic medium has been recognized for a long time. Organic compounds employed as corrosion inhibitors can adsorb on the metal surface through heteroatoms such as nitrogen, oxygen, sulphur and phosphorus. But the alcamines inhibitor has weak electromigration ability and cannot migrate to the surface of steel effectively [15]. Under high alkaline conditions, the degree of ionization of alcamines inhibitor is limited and not enough to dissociate rust, thus limiting its inhibiting effect [15]. Imidazoline inhibitors are newly trending in the field of electromigration inhibitor research. The imidazoline was reported as an effective organic corrosion inhibitor [15,16,17], and it is a novel, low toxicity and efficient water-based corrosion inhibitor which can effectively inhibit metal corrosion and be used in the neutral medium environment, as well as the acid and alkaline conditions. Wang and Shu [18] synthesized a series of imidazoline inhibitors containing with diethylenetriamine, triethylenetetramine, etc. via different processes. Kang and Li [19] used Zanthoxylum bungeanum seed oil and diethylenetriamine to synthesize an imidazoline derivative, which was then reacted with sodium chloride and benzyl chloride to obtain zwitterionic imidazoline derivatives and cationic imidazoline derivatives. Fei [20] synthesized a cationic electromigration type imidazoline inhibitor and examined its effectiveness. The methods currently used to evaluate inhibitors include the weight-loss, electrochemical, and spectral analysis methods. Both domestic and international researchers typically use electrochemical and weight-loss methods in the laboratory. Common methods employed in literature are colorimetric analysis [21], X-ray photoelectron spectroscopy [22] and surface enhanced Raman scattering [23]. Liu and Li [24] used the potentiodynamic method to study the effect of imidazoline inhibitor on the corrosion resistance of a metal surface and electrochemical impedance spectroscopy to evaluate the carbon steel spectrum for the membrane surface. These authors put forward the characteristics of the equivalent circuit. Zhang and Bai [25] studied the effect of animidazoline inhibitor using the weight-loss method and electrochemical measurements and found that the introduction of multi-adsorption centers enhanced the surfactant adsorption capacity of the metal surface, thus improving inhibition. Lei and Xiao [26] used weight-loss experiments and a self-made local corrosion simulation probe to study corrosion inhibition by imidazoline. The inhibitor was found to have an imidazoline ring structure and displayed effective inhibition. Xun and Zhou [27] used the weight-loss method to study the inhibition of corrosion by naphthenic imidazoline derivatives in sulfuric acid medium. However, imidazoline inhibitor has better protective effect on surface of metal, few researchers have studied the effectiveness of imidazoline inhibitor in concrete. Whether imidazoline inhibitor can migrate to concrete under the action of an electric field and protect the reinforcement is still unclear. In the present study, first, the objective is to develop a kind of imidazolines quaternary corrosion inhibitor for improving the corrosion inhibition, then the imidazoline and triethylenetetramine inhibitor were used for bidirectional electromigration rehabilitation (BIEM) and the effectiveness of imidazoline inhibitor in concrete was evaluated, which is also compared with triethylenetetramine inhibitor. Meanwhile, the migration of imidazoline inhibitor to concrete under an electric field and dry-wet cycle test were investigated. Finally, retention of corrosion inhibitor and long-term corrosion inhibition performance after BIEM treatment were also analyzed.

## 2. Materials and Methods

### 2.1. Materials

Sulfoxide chloride, lauric acid, NaHCO_3_, ether, ethylenediamine, triethylamine, 1,3-dibromopropane were purchased from chemical reagent factory (Ningbo, China). All of them were AR analytical reagent grade. The concrete materials include P∙O 52.5 grade cement, pebbles, medium coarse sand, and tap water.

### 2.2. Principle and Route of Imidazoline Inhibitor Synthesis

**Figure 1:**
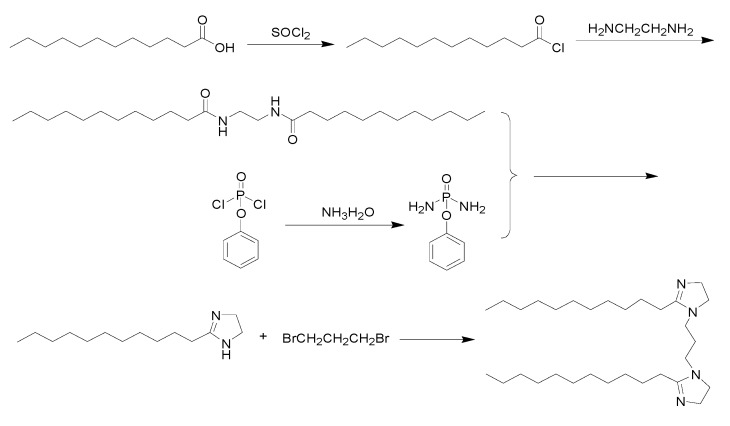
Route of new imidazoline inhibitor synthesis.

In this study, a new imidazoline inhibitor with a symmetrical molecular structure and containing two imidazoline heterocycles and two alkyl long chains of lauric acid was synthesized. The synthetic route is presented in Figure 1.

### 2.3. Synthesis of the Imidazoline Inhibitor

**Figure 2:**
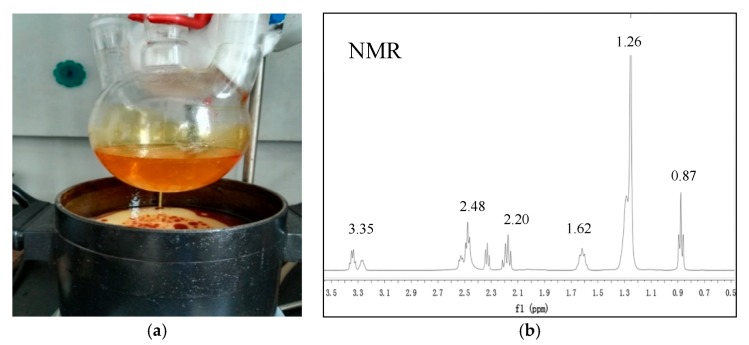
(**a**) Preparation of imidazoline inhibitor; (**b**) Characterization (^1^H NMR) of imidazoline inhibitor.

First, sulfoxide chloride dropping into lauric acid and stirring for 1–5 h at 40–80 °C. After the reaction is completed, the lauroyl chloride is obtained. Then lauroyl chloride was added to the mixed solution of water containing NaHCO_3_ and ether, and then ethylenediamine was dripped. Ethylenediamine bislauroyl amide was obtained by stirring for 6 h at 10–40 °C, filtering out the solid, washing and drying. Ethylenediamine bislauryl amide was mixed with phenoxyphosphatidyl diamide and reacted at 100–250 °C for 5–60 min. Then cooled and heated at 50–100 °C for 10–60 min. The insoluble substances were filtered and removed. Monocyclic imidazoline was obtained after cooling. Then monocyclic imidazoline lauric acid, 1,3-dibromopropane and triethylamine were dissolved in toluene and reacted for 10–25 h at 10–40 °C to obtain bicyclic imidazoline lauric acid. The synthesized imidazoline inhibitor was a light yellow solid. The solid product was easily soluble in water and had obvious surface activity, and the characterization with nuclear magnetic resonance (NMR) as shown in Figure 2. The NMR chemical shifts and peak area demonstrated the final product was imidazoline inhibitor, and δ (1.26; 0.87) is the hydrogen characteristic peak of long chain alkanes in imidazoline inhibitor, δ (1.26; 0.87) is the hydrogen characteristic peak of amide.

### 2.4. Feasibility Assessment of Imidazoline Inhibitor

**Figure 3:**
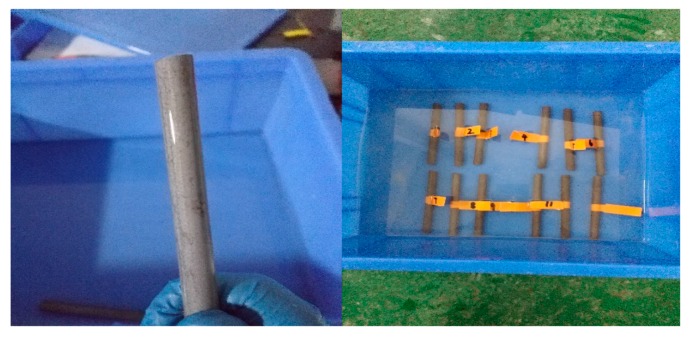
Test process of weight-loss method.

A simulating pore solution of saturated calcium hydroxide and 0.01 mol/L sodium hydroxide was used to simulate the pore fluid of reinforced concrete in Figure 3. In order to accelerate the corrosion rate, 3% sodium chloride was added to the simulated concrete pore solution.

The steel bar used in this study was an HPB235 rounded steel bar with a 10 mm (diameter) × 100 mm (length). Before the experiment, 800# sandpaper was used to remove the rust on the surface of the steel bar, which was then washed with anhydrous alcohol to remove any potentially influencing factors, such as grease. After tests on the steel bar were completed, and vacuum drying was applied after each test to avoid the distorting of further oxidation when exposed to the air. The weight was determined using a high precision electronic balance and the quality of the steel bar specimen recorded. Double ring imidazoline and triethylenetetramine were used at a concentration of 1 mol/L as inhibitors. For controls, inhibitor was not added and the remaining conditions were unchanged. Each of the 6 rust-treated specimens was placed in the inhibitor solution. After soaking for 7 and 14 days, 3 steel samples were removed from each inhibitor solution. The surface corrosion products were washed away, the steel samples were weighed after vacuum drying, and the quality of the reinforcement samples was recorded after corrosion.

In weight-loss experiments, the corrosion rate called V and corrosion inhibition rate called η calculated using Formulas (1) and (2).

Corrosion rate:

V = (m_0_ − m_1_)/s × *t*. (1)

Corrosion inhibition rate:

η = [(V_0_ − V_1_)/V_0_] × 100%. (2)

Type:

m_0_ and m_1_-quality of steel bar before and after corrosion, respectively (g)
s-Exposure area (m^2^)
*t*-Soaking time (h)
V_0_ and V_1_-corrosion rate in the absence and presence of inhibitor, respectively (g/m^2^ h)

The calculated corrosion inhibition rate results for the steel bar specimens are shown in Table 1.

**Figure 4:**
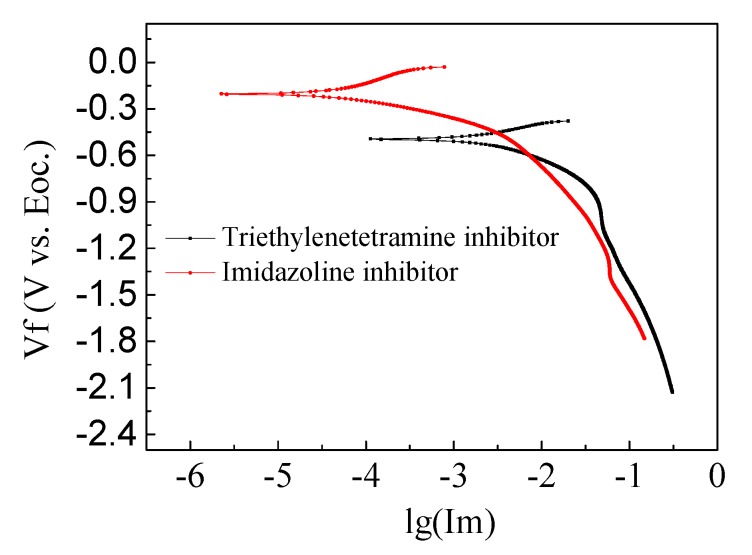
Cathodic polarization curve of stressed steel bar in corrosion inhibitor.

From the above table, it can be seen that, in the simulated concrete pore solution, triethylenetetramine had relatively less effect on corrosion of the steel bar. After 14 days of immersion, the maximum corrosion inhibition rate was 26.5%, which was far lower than that of the self-made bicyclic imidazoline inhibitor. Bicycloimidazoline inhibitor incurred good resistance to rust after 7 days immersion, where the corrosion inhibition rate reached 40.3%. After 14 days immersion, the corrosion inhibition rate reached 38.8%. The steel corrosion rate first increased and then decreased with extensions in soaking time. Based on the weight-loss method, self-made bicyclic imidazoline inhibitor has been determined to be more effective than triethylenetetramine, which can protect for steel bar corroded by chloride salt. As shown in Figure 4. The potential of steel bar in triethylenetetramine and imidazoline inhibitor solution is −0.50 V and −0.21 V, respectively, it also indicates the different corrosion resistance of steel bar.

### 2.5. Principle Underlying Bidirectional Electromigration Rehabilitation

**Figure 5:**
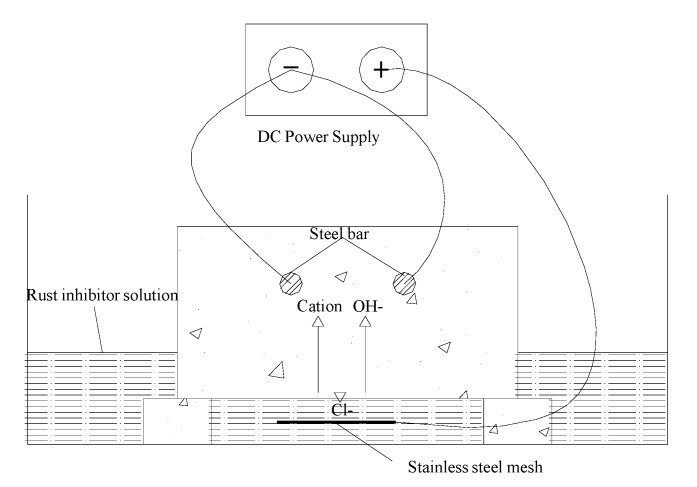
Principle underlying bidirectional electromigration rehabilitation.

The bidirectional electromigration rehabilitation device is presented in Figure 5. The bottom of the tested concrete block was soaked in a well-dispensed inhibitor solution. By applying an electrical field between the steel bar as a cathode and a stainless steel iron plate as an anode, the chloride ions and inhibitors are able to migrate out and in the concrete cover zone [28,29].

The anode and cathode reactions were as follows:

Anode reactions:

$$\begin{array}{l} 2H_{2}O\to O_{2}↑+4H^{+}+4e^{-}\\ 4{\mathrm{OH}}^{+}\to O_{2}↑+2H_{2}O+4e^{-}\\ 2{\mathrm{Cl}}^{-}\to {\mathrm{Cl}}_{2}↑+2e^{-} \end{array}$$

Cathode reactions:

$$\begin{array}{l} O_{2}+2H_{2}O+4e^{-}\to 4{\mathrm{OH}}^{-}\\ 2H_{2}O+2e^{-}\to H_{2}↑+2{\mathrm{OH}}^{-} \end{array}$$

Hydroxyl ions in the pore fluid can continue to be produced near the cathode steel bar and the steel near the original reinforced alkaline and passive corrosion can be stopped. The nitrogen ions in the inhibitor were stronger and smaller in size than the chloride ions. Therefore, the chloride ions can be isolated from the surface of the reinforcement.

### 2.6. Designing and Preparation of Concreteblocks

**Figure 6:**
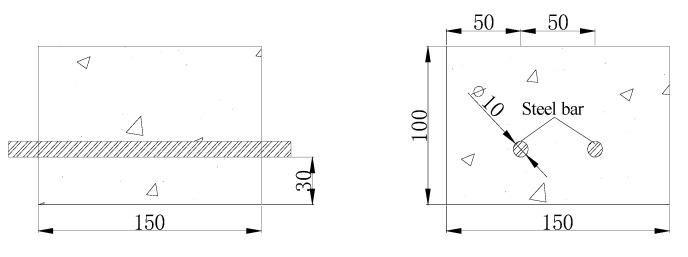
Concrete block dimensions (mm).

In this experiment, the strength grade of concrete is C30 which is according to engineering requirements. The dimensions of the concrete were 150 mm (length) × 150 mm (width) × 100 mm (height). Every concrete block contained two HPB235 round steel reinforcing bars with 10 mm diameters and the thickness of the concrete protection layer was 30 mm as shown in Figure 6. The concrete blocks were made by P∙O 52.5 grade cement, pebbles with diameters of 5.0–18.0 mm, medium coarse sand, and tap water. When pouring the concrete, a final concentration of 3% sodium chloride was added to the cementitious material to simulate a high chloride environment. The composition of the concrete block is presented in Table 2.

### 2.7. Experimental Procedure

**Figure 7:**
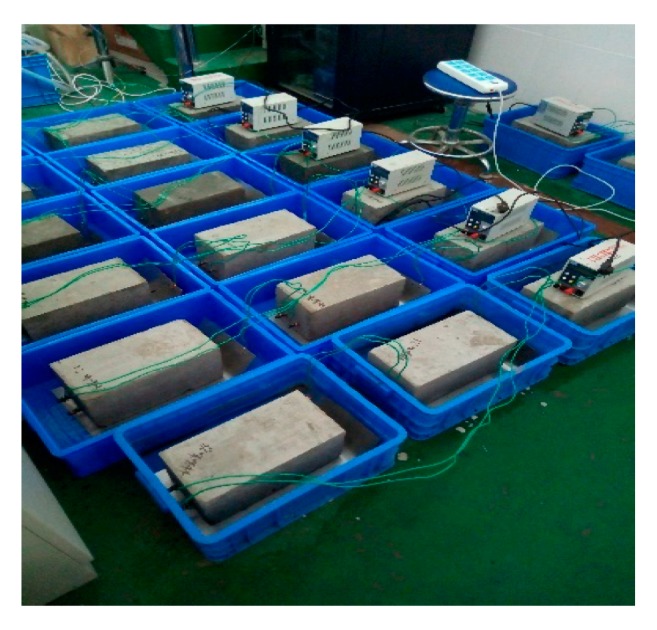
Test site.

In order to investigate the effect of the new imidazoline inhibitor on concrete corrosion resistance, experiments comparing different inhibitors were carried out. In the experiments (Figure 7), steel was used as the cathode and external barbed wire was used as the anode. The concrete was energized with a current density of 3 A/m^2^ for 14 days. The electrolytes used for bidirectional electromigration rehabilitation are listed in Table 3.

**Figure 8:**
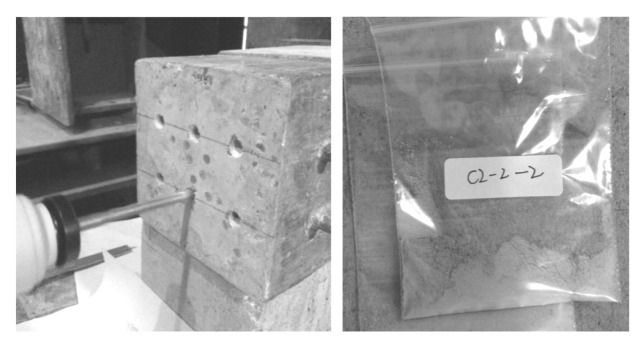
Extraction of concrete block powder.

After 14 days, the concrete block was removed, and the surface was also cleaned and then soaked for 24 h in water. After the above step, the potentiodynamic polarization curve was directly measured. Following the polarization curve test, the concrete was ventilated and dried. Based on the size of the concrete blocks, the impact drill was selected, and the concrete powder sample was taken from every 5 mm in depth (Figure 8). The concrete powder was sifted through a mesh screen with a mesh diameter of 0.3 mm and immersed in deionized water for 24 h and then the chloride ion content in the deionized water was determined. The inhibitor content in the concrete was also measured based on the nitrogen content. The concrete powder sample was collected from every 10 mm in depth. Each test sample required 20 mg concrete power with particles sized > 80 μm to test the inhibitor content. Therefore, the concrete powder was sifted through mesh screen with a mesh diameter of 0.075 mm and concrete powder with particle sizes > 0.075 mm was stored in centrifuge tubes.

After the concrete powder samples were retrieved from the C30, the test pieces were broken and cement hydration products near the surface of steel bar with diameters of no more than 10 mm were retrieved for analysis by scanning electron microscopy. After the concrete powder sample was collected from the concrete samples, the axial compressive strengths of the concrete blocks were determined. And the specimens were obtained near anode and cathode with different charging times (concrete of type 1, current density: 3 A/m^2^, 3% NaCl, charging time: 7 days, 15 days, and 30 days) using a mercury porosimeter. The Hg intrusion method was introduced to measure the porosity.

### 2.8. Dry-Wet Cycle Test of Concrete with Imidazoline Inhibitors

**Figure 9:**
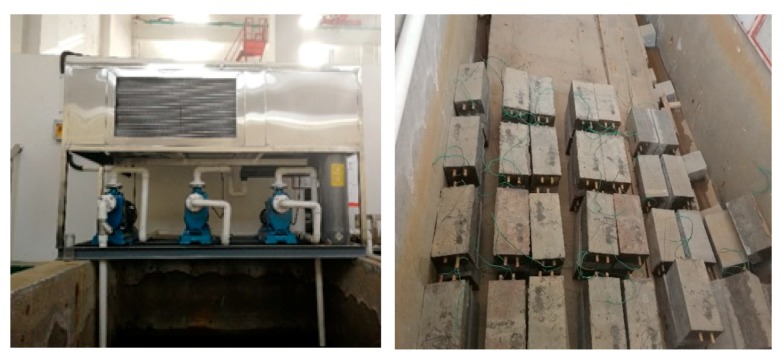
Drying-Wetting Cycles test.

By adding 0.25%, 0.5% and 0.75% imidazoline inhibitors into C30 concrete, and BIEM is introduced during the curing period. The current density is set to 3 A/m^2^ and the power-on time is 15 days, and the samples were marked as Table 4. After the power-on, the dry-wet cycle test (Figure 9) was initiated at the same time. The concentration of sodium chloride in tidal simulator is 3.5%. 72 h as a cycle of drying and wetting in the experiment. In each cycle, concrete specimens were soaked for 18 h and dried for 54 h. The dry-wet cycle time of reinforced concrete specimens is 30 days.

## 3. Results and Discussion

### 3.1. Corrosion Potential and Resistance of Steel

In previous research, we have proposed the remedial technique of bidirectional electromigration rehabilitation (BIEM), through which chloride (Cl^−^) ions are successively removed along with the injection of inhibitors into the ordinary reinforced-concrete specimens [28,29]. Triethylenetetramine (TETA), an amine-based inhibitor, was used during the electrochemical process. Nevertheless, the electrochemical migration capabilities of triethylenetetramine inhibitor was found to be lower than expectation. The new imidazoline inhibitor with a different structure from the traditional inhibitor is thus investigated. In the following experiment, the corrosion potential, and concentration profiles of Cl^−^ ions were measured before and after the electrochemical process. According to the specification for inhibitors (GB/T 33803-2017), when the potential of steel is between −350 and −500 mV, the probability of steel corrosion is 95%, and if the potential of steel is higher than −200 mV, the probability of steel corrosion is less than 5%.

**Figure 10:**
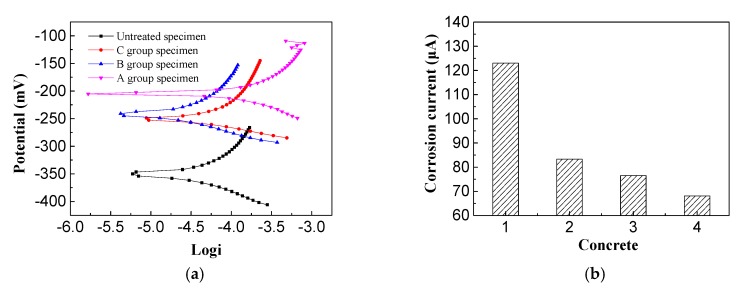
Corrosion potential and current of steel bar. (**a**) Corrosion potential; (**b**) Corrosion current.

From Figure 10a, it can be seen that the corrosion potential of the untreated reinforced concrete specimens was the most negative, reaching −350 mV, and the corrosion potentials of the reinforced concrete specimens after electrochemical repair were all moving forward. The most positive shift in corrosion potential (145 mV) was found in the imidazoline-treated sample, which is 107.3 mV for the triethylenetetramine-treated one and 100 mV for the electrochemical chloride extraction (ECE) treated one. In general, more positive corrosion-resistance potential of the steel indicates a more extensive area of passivation, which is beneficial to the durability of the reinforced concrete. Based on the positive shift in corrosion potential, the imidazoline and triethylenetetramine electromigration inhibitors promoted the durability of concrete. From the steel corrosion potential amplitude, it can be seen the imidazoline has a better performation in preventing corrosion than triethylenetetramine, and triethylenetetramine is a better inhibitor of rust than electrochemical chloride extraction.

Figure 10b presents the analysis performed using reinforcement corrosion current software with the electrochemical workstation, where 1, 2, 3, and 4 represent the reinforced corrosion currents of the untreated, electrochemical chloride extraction-treated, triethylenetetramine-treated, and imidazoline-treated reinforced concrete specimens, respectively. It can be seen that the corrosion current of the untreated reinforced concrete specimens was the largest at 123 μA, while the corrosion current of the imidazoline-treated specimens was the smallest at 68.1 μA. The corrosion current of the triethylenetetramine-treated samples was 76.5 μA and the corrosion current of the electrochemical chlorination-treated samples was 83.3 μA. The corrosion current of the reinforcement reflects the corrosion rate of the steel bar under no external current. The corrosion rate is proportional to the corrosion current, where the larger the corrosion current, the faster the corrosion rate. Imidazoline and triethylenetetramine both reduced the corrosion current and slowed down the corrosion rate of the steel bar and protected the durability of the concrete structure. The corrosion current of the imidazoline was higher than that of triethylenetetramine, indicating imidazoline was more effective than triethylenetetramine.

Based on the above analysis, imidazoline and triethylenetetramine inhibitor both delay corrosion, and increase corrosion resistance of steel bars in concrete, where the imidazoline inhibitor is more effective overall than triethylenetetramine, the main reason is many nitrogen adsorption centers on the imidazole heterocycle, and it has strong ability to combine with metal and form a more stable structure.

### 3.2. Chloride Ion Concentrations in Concrete

**Figure 11:**
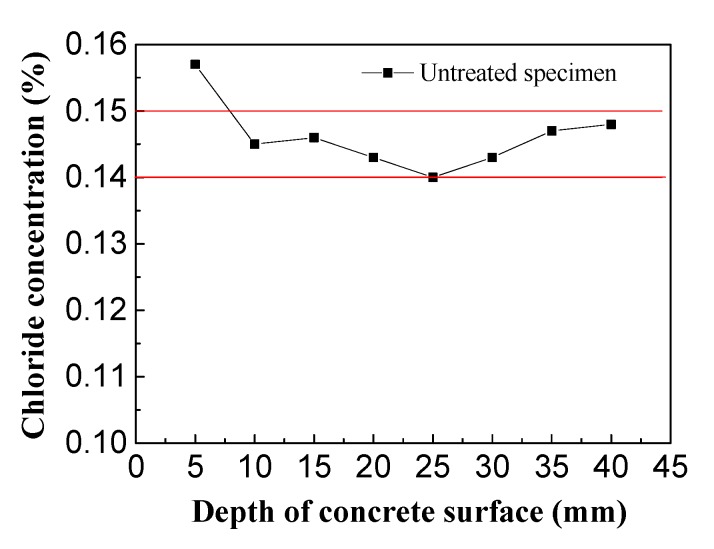
Initial chloride concentration in concrete.

The control group consisted of concrete specimens not treated by electrochemical rehabilitation and was used to determine the initial chlorine ion concentrations in the chloride-corroded concrete (Figure 11).

From the above chart, it can be seen that the chloride ion concentrations in relation to the concrete protective layer thickness ranged from 0.14%–0.15% with small fluctuations, indicating the concrete protective layer was evenly distributed along the initial direction of chloride ions and the initial concrete chloride concentration was 0.1446%. In the experiment, the electrolytes were imidazoline solution, triethylenetetramine solution and saturated calcium hydroxide solution. The residual chloride ion distribution in the concrete specimens was detected after electrochemical treatment, and the effects of imidazoline and triethylenetetramine on chloride ion migration were studied.

**Figure 12:**
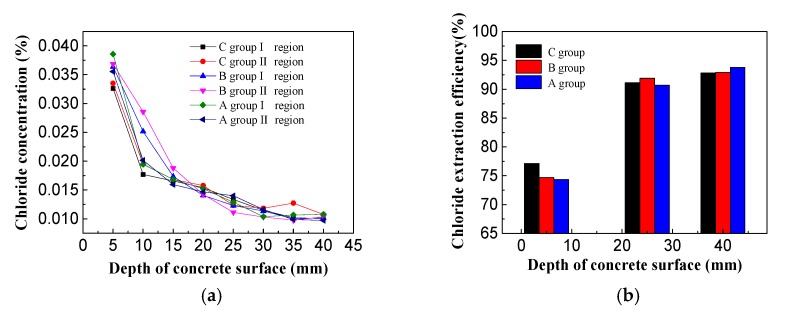
(**a**) Chloride concentration of concrete specimens; (**b**) Chloride extraction efficiency of concrete specimens.

After the electricity was completed, three holes were taken under the area of the steel bar. The chloride concentration was measured by Chloride-Meter DY-2501 and the chlorine ion discharge efficiency was calculated (Figure 12).

As shown in Figure 12a, it can be seen that the residual chloride concentrations in the specimens treated with the three different electrolytes all displayed a decreasing trend along the thickness of the protective layer. Overall, the residual chloride ion concentration was highest on the surface of the concrete and lowest on the steel bar. No obvious differences were observed in the internal residual chloride levels in the concrete specimens for the three electrolytes and the residual chloride concentrations at the steel bar were about 0.01%. From the above diagram, it can be found that there were slower changes in and more stable levels of residual chloride ions closer to the steel bar. On the surface of concrete specimens, the residual chloride ion concentration of saturated calcium hydroxide solution is the lowest. The residual chloride ion concentration of I region is 0.0326%, and that of II region is 0.0335%. The residual chloride ion concentration of imidazoline solution and triethylenetetramine solution on the surface of steel bar specimens is slightly higher than that of saturated hydrogen. In calcium oxide solution, the residual chloride ion concentration ranges from 0.035% to 0.040%. The residual chloride ion concentration on the surface of concrete specimens after electrochemical treatment is the highest, but it is much lower than 0.1% required by the code. From the Figure above, it can be seen that the closer to the reinforcement, the more stable of residual chloride ion concentration, while near the surface of concrete specimens, the greater change of residual chloride ion concentration, which is related to the distribution of electric field inside concrete [30,31].

In Figure 12b, the chloride extraction efficiency diagram for specimens electrochemically treated with different electrolytes at 5, 25, and 40 mm from the surface of the concrete. The chloride extraction efficiency was lowest closer to the surface (5 mm), where the efficiencies of the three electrolyte treatments ranged from 70%–80%. The removal efficiency of the chlorine-saturated calcium hydroxide solution was slightly higher than the other two solutions tested. The averaged chloride extraction efficiency of both the imidazoline solution and triethylenetetramine solution on the surface of the concrete are close to 77%, which displayed no obvious difference. The chloride extraction efficiencies of the three electrolyte solutions at 25 and 40 mm were higher than 90%. The chloride extraction efficiency of triethylenetetramine was slightly higher than the other two treatments at 25 mm and the chlorine removal efficiency was 91.9%. The chloride extraction efficiency of imidazoline was slightly higher than that of the other two treatments at 40 mm and the chloride extraction efficiency was 93.8%. There were no obvious differences in the efficiency of chlorine removal by the three electrolyte solutions.

Based on the above analysis, it can be concluded that imidazoline and triethylenetetramine were excellent electromigration inhibitors after 15 days electricity treatment. Most of the chloride ions migrated out of the concrete specimens, which is conducive to improving the durability of concrete structures. There were no significant differences in residual chlorine concentration and chloride removal efficiency between the two inhibitors and the addition of inhibitors only in the saturated calcium hydroxide solution. Therefore, the electromigration inhibitor does not affect the migration of chloride ions.

### 3.3. Inhibitor Concentrations in Depth of Concrete

**Figure 13:**
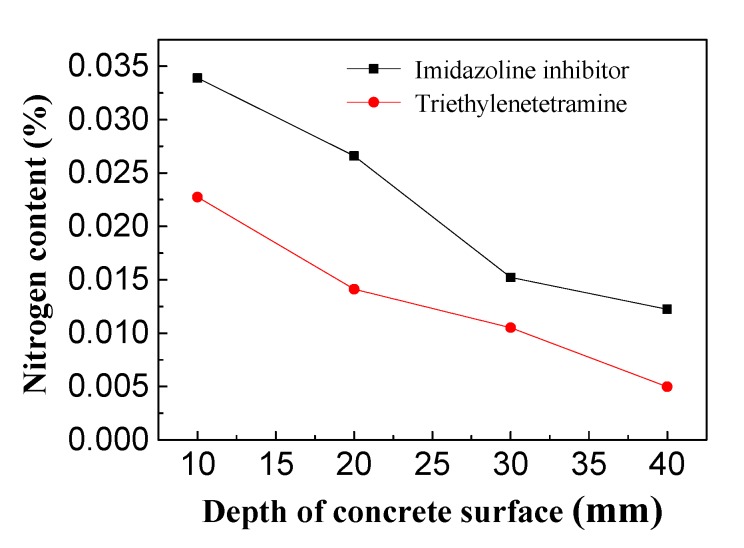
Inhibitor content in concrete.

It is known that organic corrosion inhibitors are effective in preventing steel from corroding, and the concentration of the inhibitors is crucial to provide adequate protection for the reinforcement [32,33,34]. As shown in Figure 13, the inhibitor concentration in the reinforced concrete decreased as the depth from the concrete surface increased. Specifically, the inhibitor content was highest on the surface of the concrete and lowest on the steel bar. The highest and lowest concentrations of imidazoline inhibitor in the concrete specimens were approximately 0.035% and 0.013%, and the highest and lowest content of triethylenetetramine were 0.023% and 0.005% in concrete, respectively. The amount of imidazoline inhibitor in the concrete was higher than triethylenetetramine. The surface inhibitor content in concrete was about three-fold that of triethylenetetramine and the content of reinforcing bar inhibitor was 2.6-fold that of triethylenetetramine. For triethylenetetramine and electrochemical chloride extraction, the corrosion potential and current of the concrete bar were very similar. This may be because the triethylenetetramine content on the surface of steel bar was very low and with less effective at rust protection for the reinforcing steel bar. In the experiments, the same concentration of imidazoline and triethylenetetramine were used with 0.3 mol/L, after treatment, the imidazoline inhibitor content in the concrete was higher than that of triethylenetetramine, which indicating that the imidazoline experienced stronger migration under the electric field than the triethylenetetramine.

### 3.4. Potential of Reinforcement Bar after Dry-Wet Cycle

**Figure 14:**
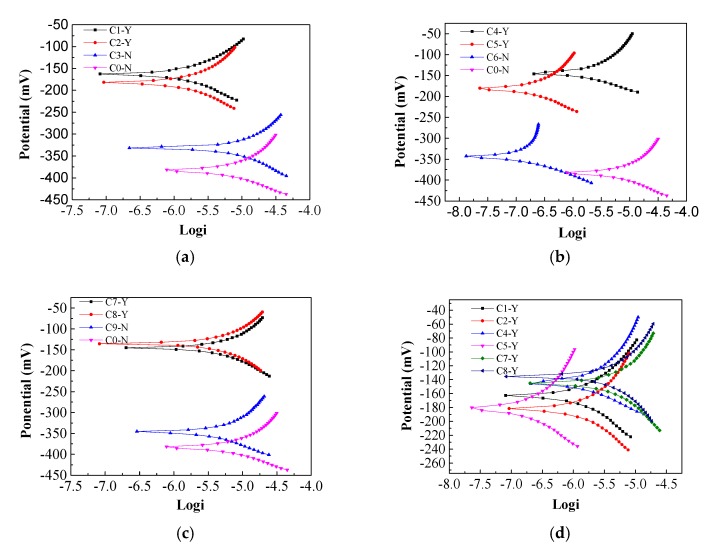
Corrosion potential of reinforcement bar. (**a**) 0.25% imidazoline inhibitor; (**b**) 0.5% imidazoline inhibitor; (**c**) 0.75% imidazoline inhibitor; (**d**) imidazoline inhibitor.

From Figure 14, it can be seen that the corrosion potential of concrete C0-N (power-off) is about −380 mV. During the experiment, the current density is set to 3 A/m^2^ and the power-on time is 15 days, after the power-on, the dry-wet cycle test initiated at the same time. In each cycle, concrete specimens were soaked for 18 h and dried for 54 h. From Figure 14a, after electrification treatment, the potential of concrete C1-Y (power-on) ranges from −180 mV to −150 mV, which increased about 200 mV than that of C0-N, and from Figure 14b, it can be seen that the potential of concrete C2-Y ranges from −180 mV to −130 mV, which also increased by about 200 mV more than that of C0-N, and while it increased imidazoline inhibitor in concrete, it can also be learned from Figure 14c, the potential of concrete C3-Y ranges from −150 mV to −130 mV, which increased about 230 mV than that of C0-N, the above results show that the corrosion resistance of concrete can be improved by mixing different concentration of imidazoline inhibitor.

### 3.5. Characteristics of Chloride Ion Diffusion in Concrete

**Figure 15:**
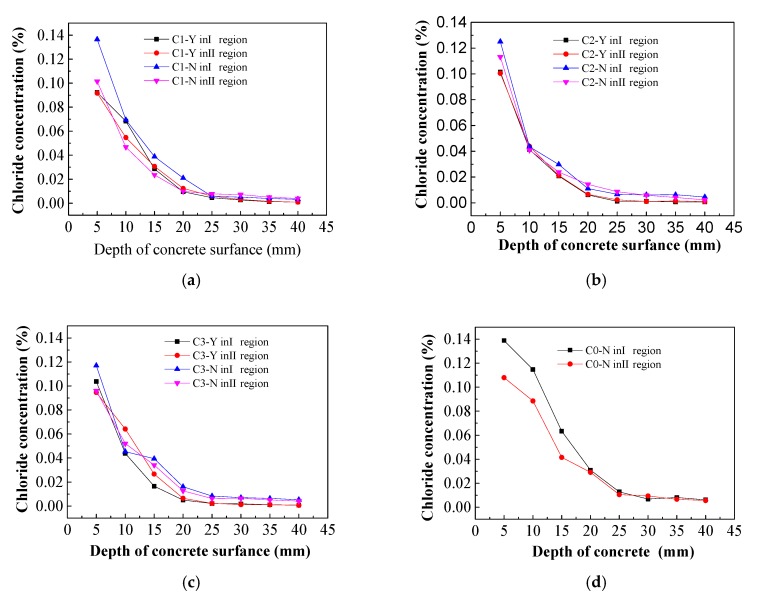
Chloride concentration. (**a**) 0.25% imidazoline inhibitor; (**b**) 0.5% imidazoline inhibitor; (**c**) 0.75% imidazoline inhibitor; (**d**) Control samples.

From Figure 15, in different regions of concrete specimens, it can be seen that the chloride ion concentration of concrete specimens with imidazoline inhibitor decreases along the thickness direction of the protective layer after dry-wet cycle [35,36]. The chloride ion concentration on the surface of concrete specimens is the highest and the chloride ion concentration on the reinforcement is the lowest. The concentration of chloride ion varies greatly from 0 to 25 mm away from the surface of concrete specimens, while the concentration of chloride ion varies slightly from 25 to 40 mm, which tends to be stable, it showed that the area of chloride ion erosion is more obvious from the surface of concrete to the area of 25 mm away from the surface of concrete [37,38]. The chloride ion concentration distribution trend of control specimens is the same as that of concrete specimens with imidazoline inhibitor. The chloride ion concentration of concrete surface is about 0.1%, but the chloride ion concentration of steel bar in C0-Y is higher than that of concrete specimens with imidazoline inhibitor.

### 3.6. Hydration Productanalysis of Treated Samples

**Figure 16:**
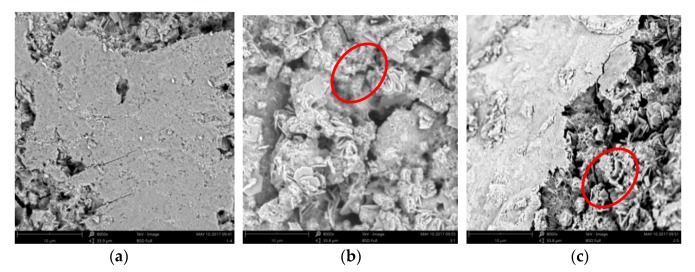
Scanning electron microscope (SEM) images of different samples. (**a**) Reference sample; (**b**) electrochemical chloride extraction (ECE) treated sample; (**c**) imidazoline inhibitor (IM) treated sample.

Figure 16 shows that in the reference sample (untreated), the hydration product contained a large amount of needle-shaped ettringite, calcium hydroxide, and more lamellar silicate gel C-S-H. In electrochemical chloride extraction (ECE) treated sample, the needle-shaped ettringite were virtually invisible, but there was a large amount of stacked lamellar calcium hydroxide and comparatively less lamellar silicate gel C-S-H. In the imidazoline inhibitor (IM) treated sample, which consisted of samples treated with bidirectional electromigration rehabilitation, there was an increasing in flake calcium hydroxide near the reinforcement, which is very beneficial for protecting the steel bars. However, the hydration product density of the concrete decreased and the porosity increased in the ECE and IM samples, perhaps due to the production and outward diffusion of hydrogen during energization [39,40]. Conversely, lamellar silicate gel C-S-H hydrolysis by electricity may lead to expansion of the pores.

### 3.7. Pore Distribution of BIEM Treated Samples

**Figure 17:**
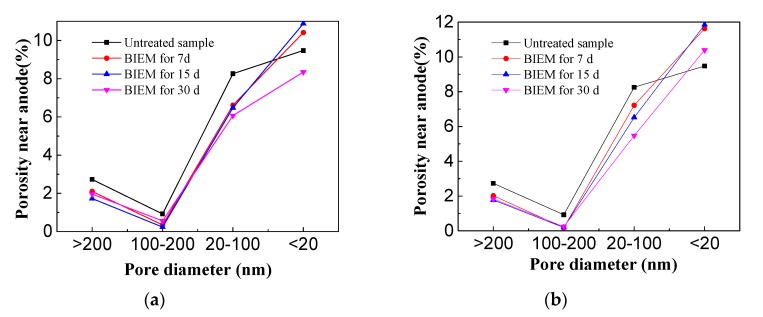
Porosity distribution of concrete cover after Bidirectional electromigration rehabilitation (**a**) Anode side; (**b**) Cathode side.

As shown in Table 5, and Figure 17, the Porosity of concrete decreased after BIEM treatment. Wu [41] classified the pore diameters into the following grading (1973): Great harmful pore (>200 nm), harmful pore (100–200 nm), little harmful pore (20–100 nm), and harmless pore (<20 nm), Figure 17a,b shows the pore distributions of concretes near the anode and cathode, respectively, while the pore below 20 nm, the porosity increased with BIEM treatment for 7 days and 15 days. And the porosity of harmless pore (<20 nm) with BIEM treatment with 15 days is higher than that of 7 days, but when the charging time increases to 30 days, the porosity of harmless pore near the cathode is lower than that of 15 days, while the porosity of harmless pore near the anode is lower than that of untreated sample. The above results are due to the process of BIEM treatment, the hydration products decomposed and ions dissolved in pore of concrete cover, then pores with small diameters formed [29], Furthermore, water electrolysis will generate hydrogen ions whose formation of gaseous hydrogen will cause increase in porosity near the reinforcement [42,43]. Thus, the porosity of the concrete is higher near the cathode than near the anode.

## 4. Conclusions

In the present study, the effectiveness of self-made imidazoline inhibitor on the durability of reinforced concrete was sysmetically investigated and compared with the conventional triethylenetetramine inhibitor. It can be concluded that:

(1) The effect of electrochemical treatment with different inhibitors on chlorine salt erosion of reinforced concrete corrosion potential and corrosion current were studied. It was found that imidazoline and triethylenetetramine inhibitors can increase the corrosion potential to some extent. The imidazoline corrosion potential shifted positively to the amplitude ratio of triethylenetetramine. The two inhibitors reduced the corrosion current, where the imidazoline corrosion current displayed a more significant reduction than triethylenetetramine.
(2) Inhibitor from the external solution was found to discharge the majority of chloride ions in the concrete. The chloride extraction effect and electrochemical chloride extraction made no significant difference on the surface of the concrete, where chlorine removal efficiency and the efficiency of chlorine has reached the requirement around the location of steel, which revealing that the electromigration inhibitor did not affect the migration of chloride ions in the concrete.
(3) The inhibitor content in the concrete specimens was evaluated after BIEM. After a dry-wet cycle test, the potential of the concrete increased by about 200 mV by mixing the imidazoline inhibitor, and the inhibitor content in the concrete specimens decreased along the thickness of the concrete protective layer. The imidazoline inhibitor content in the concrete was higher than that of triethylenetetramine, which indicates that the electric migration ability of the imidazoline inhibitor was stronger than the other compounds under an electric field.

## Figures and Tables

**Table 1:** Corrosion inhibition rates of different inhibitors in simulated pore fluid of concrete.

Test Conditions	Inhibitor	Time (d)	Mass Loss (g) 9	Rate
Pore solution	-	7	0.0710	-
		14	0.1499	-
Pore solution	Triethylenetetramine	7	0.0585	17.6%
		14	0.1101	26.5%
Pore solution	Imidazoline	7	0.0424	40.3%
		14	0.0916	38.8%

**Table 2:** Mixture proportions.

Strength	Water (kg/m^3^)	Cement (kg/m^3^)	Sand (kg/m^3^)	Gravel (kg/m^3^)
C30	177	393	534	1297

**Table 3:** Samples of electrolytes.

Samples	Strength	Electrolyte	
		New Imidazoline	TETA
A	C30	0.3 mol/L	-
B		-	0.3 mol/L
C		-	-

**Table 4:** Test setting of reinforced concrete.

Samples	Inhibitor	Amount of Inhibitor	Power (Y/N)
C0-N	Imidazoline	0	N
C1-Y		0.25%	Y
C2-Y		0.25%	Y
C3-N		0.25%	N
C4-Y		0.5%	Y
C5-Y		0.5%	Y
C6-N		0.5%	N
C7-Y		0.75%	Y
C8-Y		0.75%	Y
C9-N		0.75%	N

**Table 5:** Porosity changes after BIEM treatment.

	Time	0	7 Days	15 Days	30 Days
Position					
Porosity near anode		21.39%	19.47%	19.32%	16.93%
Porosity near cathode			21.04%	20.33%	17.93%
